# Texture and Differential Stress Development in W/Ni-Co Composite after Rotary Swaging

**DOI:** 10.3390/ma13122869

**Published:** 2020-06-26

**Authors:** Pavel Strunz, Radim Kocich, David Canelo-Yubero, Adéla Macháčková, Přemysl Beran, Ludmila Krátká

**Affiliations:** 1 Nuclear Physics Institute of the CAS, Řež 130, 25068 Řež, Czech Republic; caneloyubero@ujf.cas.cz (D.C.-Y.); premysl.beran@esss.se (P.B.); 2 Faculty of Materials Science and Technology, VŠB-Technical University of Ostrava, 708 00 Ostrava, Czech Republic; radim.kocich@vsb.cz (R.K.); adela.machackova@vsb.cz (A.M.); ludmila.kratka@vsb.cz (L.K.); 3 ESS (European Spallation Source ERIC), SE-221 00 Lund, Sweden

**Keywords:** tungsten heavy alloys, rotary swaging, neutron diffraction, residual stress, texture

## Abstract

Knowledge of texture and residual stresses in tungsten heavy pseudoalloys is substantial for the microstructure optimization. These characteristics were determined in cold and warm rotary swaged W/NiCo composite with help of neutron diffraction. The results were discussed in view of the observed microstructure and mechanical properties. The investigated bars consisted of tungsten agglomerates (bcc lattice) surrounded by NiCo-based matrix (fcc lattice). No preferential crystallographic orientation was found in the as-sintered bar. A strong texture was formed in both the tungsten agglomerates (<101> fiber texture parallel to the swaging axis) and in the NiCo-based matrix (<111> fiber texture) after rotary swaging. Although usually of double-fiber texture, the <001> fiber of the fcc structures was nearly missing in the matrix. Further, the cold-swaged bar exhibited substantially stronger texture for both phases which corresponds to the higher measured ultimate tensile strength. The residual stress differences were employed for characterization of the stress state of the bars. The largest residual stress difference (≈400 MPa) was found at the center of the bar deformed at room temperature. The hoop stresses were non-symmetrical with respect to the swaging axis, which was likely caused by the elliptical cross section of the as-sintered bar.

## 1. Introduction

Tungsten heavy alloys (THAs), typically used in challenging applications such as aircraft counter-balances, gyroscope rotors, radiation shields, and kinetic penetrators in the military industry, exhibit excellent mechanical and physical properties [1]. Given their microstructure, typically containing approximately spherical tungsten agglomerates (usually 90–97 wt%) embedded in a ductile matrix consisting of other elements (e.g., Co, Ni, Fe, and Cu), THAs are also termed as composites or pseudoalloys [2,3]. Due to the substantial difference between the melting temperatures of tungsten and matrix-forming elements, THAs can hardly be cast and thus the typical fabrication technology of THAs combines methods of powder metallurgy (fabrication of powders—preferably by mechanical alloying, subsequent sintering, and possible final quenching), and processing via plastic deformation under hot or cold conditions [4]. Since the final performance and properties of the product can significantly be influenced by the selected processing steps, it is advantageous to optimize the used technology with help of characterization of the structural and microstructural phenomena (such as possible presence of residual stress and texture) and their effects on the final properties for each THA chemical composition and intended final product application [5,6].

A fundamental parameter having the most substantial influence on the structure characteristics and mechanical properties is the grain size. This parameter can be affected during the final production steps involving processing via plastic deformation. Effective grain refinement can be advantageously introduced by applying techniques imposing intensive shear strain, such as the methods of severe plastic deformation (SPD). Several research works showed that imposing intensive shear strain into THAs during their production enhances their utility properties and ballistic performance [7,8]. Probably the most widespread SPD method is the equal channel angular pressing (ECAP) and its modifications (TCAP [9], TCMAP [10], NECAP [11], etc.), which have been proven to refine effectively the grain size to the ultra-fine-grained (UFG) scale for various materials ranging from aluminium [12], through magnesium [13], copper [14], steel [15], and also tungsten [16,17]. However, ECAP is a discontinuous process, as the majority of SPD methods including the most effective methods of high-pressure torsion (HPT) [18] are, and is only suitable for processing samples of finite dimensions. This disadvantage limits a prospective industrial scale-up of conventional SPD methods for THAs production. Primarily for this reason, the currently used methods of THAs fabrication are mostly based on conventional processing technologies, such as hot extrusion [19,20], cold rolling [21], and combinations of thermomechanical processing and ageing [22], although studies documenting positive effects of SPD methods on improvement of THAs performance were reported [23,24,25].

Nevertheless, industrially applicable methods of imposing intensive shear strain into the processed materials exist as well. Rotary swaging (RS) is, amongst the intensive plastic deformation methods, advantageously used in the industry, primarily in the automotive, to manufacture solid, hollow and/or shaped axisymmetric products via gradually reducing cross-sections and increasing lengths of the processed workpieces [26]. The technology incrementally imposes substantial shear strain into the processed material and thus provides significant plastic deformation resulting in the elimination of residual porosity in the microstructure refinement and in the enhancement of mechanical and utility properties [27]. RS is not only suitable for processing of conventionally cast alloys but can also be favorably used to process composites, pseudoalloys, and sintered materials [28].

During the plastic deformation, the slip process induces crystal lattice rotation. In polycrystalline metals, this re-orientation of grains develops the deformation texture [29]. In many materials, properties are texture specific. Plastic deformation also induces residual stress into the deformed component. Both texture and residual stress are important microstructural features which influence the final mechanical properties for THAs. Material properties such as strength, stress corrosion cracking resistance, deformation behavior or resistance to radiation damage can be highly dependent on the texture and related changes in the microstructure. It is, therefore, important to know both the texture and residual stress. The process of a component fabrication can be then optimized.

One of the techniques beneficially used in studying structure and microstructure of metallic materials is neutron diffraction [30]. One of the main advantages of this method lies in the possibility to acquire data from the bulk of the sample, not only from its near-surface regions. This is crucial especially for THAs mainly consisting of tungsten, which is highly absorbent for other radiation types (X-ray, electrons). Although tungsten is also relatively highly absorbent also for neutrons, neutron path length through the alloy up to 15 mm is still possible even from medium flux neutron sources. Utilization of neutrons enables the signal to be averaged over a relatively large volume and the effects of local variability, large grain size, and possible local artefacts are thus minimized. The large penetration capability of thermal neutrons in most metallic materials allows nondestructive measurements of both texture and elastic strains/stresses [31] within the bulk of large polycrystalline specimens.

The primary aim of the presented study is to determine texture and differential residual stress in the investigated THA after sintering, as well as after processing via rotary swaging at various temperatures. The study is mainly based on a characterization of the occurring microstructural phenomena via neutron diffraction.

## 2. Materials and Methods

The W, Ni and Co powders with the initial particle sizes ranging between 2 and 4 µm were at first homogeneously mixed for 24 h to create the 93W-6Ni-1Co mixture in wt.%. The initial powder mixture is depicted in the next section. The mixture was subsequently compressed via cold isostatic pressing at 400 MPa into bars, which were then sintered at 1500 °C under H_2_ protective atmosphere. After sintering, the bars with approximately 12 × 18 mm^2^ elliptical cross sections were quenched in water. Throughout the following text, the green as-sintered material is denoted as W_0. The sintered bars were subsequently processed via rotary swaging either at room temperature (sample W_A), or at 900 °C (sample W_B). The first swaging pass was essential to transform the elliptical bar cross-section to a circular diameter. The following swaging passes were then performed to achieve the final circular swaged bars with the diameter of 10 mm.

The microstructures of the sintered and swaged bars were observed on polished transversal cross-sectional cuts acquired by electro-erosive cutting. Back-scattered electron (BSE) analysis in scanning electron microscopy (SEM) using Tescan Lyra 3 device (Tescan Brno s.r.o, Brno, Czech Republic) was used.

For texture determination, the neutron diffraction patterns were collected using the MEREDIT diffractometer of CANAM infrastructure at NPI Řež near Prague [32]. A mosaic Cu monochromator (reflection 220) was used to provide neutrons with the wavelength of *λ* = 0.146 nm. The neutron beam cross section was 10 × 10 mm^2^. The multi-detector bank (35 × ^3^He point counters with corresponding 10′ Soller collimators) was positioned to four different angular positions in order to collect intensities of altogether four different reflections at different *2θ* angles (*θ* being the scattering angle). The samples for the texture determination were fixed in the beam using a goniometer enabling their angular positioning. Intensities of the reflections were angularly scanned for both present phases within all the three sample bars in order to reveal their textures. The angular steps of the sample tilt and of the sample rotation was 5°.

**Figure 1:**
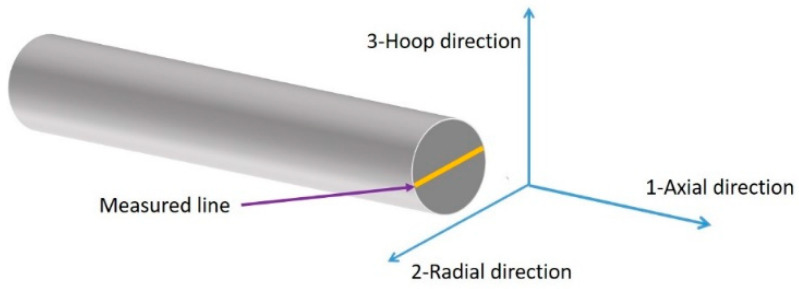
Sketch of the sample indicating the three orthogonal strain directions: axial (z-direction, denoted also as (1), radial (2) and hoop (3).

Bulk-average elastic strain/stress measurement using neutron diffraction is based on the determination of *d_hkl_* interplanar distance for the selected crystallographic plane. For strain determination, the experiment was performed using the SPN-100 neutron diffractometer installed at the LVR-15 research reactor in Řež [33]. This experimental facility is dedicated for mapping of residual strains in polycrystalline materials. SPN-100 instrument is equipped with curved Si monochromator and position-sensitive detector (PSD) for fast recording of diffraction patterns. Neutron wavelength of *λ* = 0.213 nm and the tungsten 110 reflection were selected for the measurements leading to the diffraction angle *2θ* of approximately 60°. This is not an optimum geometrical diffraction arrangement (which would be at *2θ* ≈ 90° with an irradiated gauge volume of a cubic or rectangular shape). Nevertheless, selection of *2θ* = 90° would have resulted in studying the (200) crystallographic plane that is strongly affected by intergranular strains, i.e., large difference with macroscopic residual stresses. The incident beam was defined by 2 × 17 mm^2^ and 2 × 2 mm^2^ cadmium slits for the hoop/radial and axial strains, respectively. The cadmium slit of 2 mm width was used for the diffracted beam for all the strains. The *2θ* angle determined in the diffraction experiment was then averaged over the elongated gauge volume of ½ × (4 × 2.3 × 17) mm^3^ and ½ × (4 × 2.3 × 2) mm^3^ for the hoop/radial and axial strains, respectively. To determine the *2θ* angles in all three directions, different geometrical arrangements of the examined sample with respect to the scattering vector were performed [34]. The line measured during the experiment is shown in Figure 1. Only the positions up to ±3 mm from the center were measured to ensure the gauge volume to be completely within the sample, avoiding spurious strains (remind, that the sample diameter was 10 mm).

The sintered bar was originally considered as the *d*_0_ sample. However, this stress-free reference was later dismissed for three main reasons:

- The tungsten heavy alloy is formed by two phases, W and NiCo, that provoke thermal mismatch between them, aggravating complete relaxation.
- During cooling from the sintering temperature, macro-stresses are generated within the bulk of the sample, varying the interplanar distance.
- During rotary swaging, mainly at high temperature, the phases within the sample can undergo inter-diffusion, hindering the comparison with the sintered sample.

When the *d*_0_ reference is not available, an alternative method described by Graces [35] can be used to calculate the stress differences in three orthogonal directions:

(1) $$\sigma_{1}-\sigma_{3}=-\frac{E}{\left(1+\nu \right)}\left(\theta_{1}-\theta_{3}\right)cot\theta_{M}$$

(2) $$\sigma_{2}-\sigma_{3}=-\frac{E}{\left(1+\nu \right)}\left(\theta_{2}-\theta_{3}\right)cot\theta_{M}$$

(3) $$\sigma_{1}-\sigma_{2}=-\frac{E}{\left(1+\nu \right)}\left[\left(\theta_{1}-\theta_{3}\right)-\left(\theta_{2}-\theta_{3}\right)\right]cot\theta_{M}$$

where *θ*_1_, *θ*_2_ and *θ*_3_ are the measured *θ* angles in directions 1, 2 and 3, respectively, and *θ_M_* corresponds to an arbitrary fixed value in the vicinity of the unknown value *θ*_0_. These expressions do not require any *d*_0_ of a stress-free reference.

Mechanical properties of the investigated bars were tested by tensile tests. The testing was performed using a Zwick/Roel device (ZwickRoell Czech Republic, Brno, Czech Republic) with bars of 100 mm length at the strain rate of 1.3 × 10^−3^ s^−1^. The measured stress-strain curves of the as-sintered and swaged material states were published previously in [36].

## 3. Results

### 3.1. Structure Characterization

**Figure 2:**
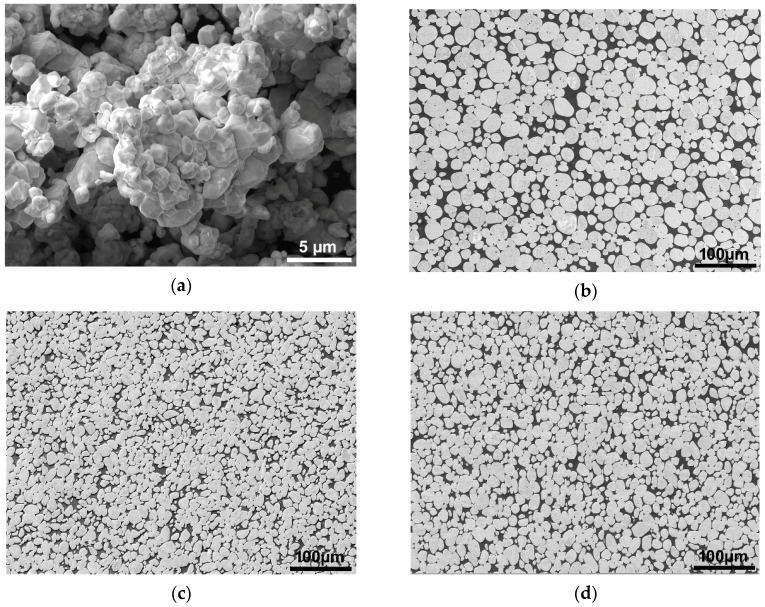
Original powder mixture (**a**); structures of investigated sample bars: W_0 (**b**); W_A (**c**); W_B (**d**).

Figure 2a depicts an image of the original powder mixture, whereas structures of the W_0, W_A, and W_B sample bars are depicted in Figure 2b–d, respectively. As can be seen, the powder mixture consolidated sufficiently during sintering and the structure of the sintered and quenched W_0 sample consisted of approximately spherical tungsten agglomerates surrounded by the NiCo-based matrix (Figure 2b). Based on our previous study [36], the phases are the *α*-W phase with B2 structure (bcc), further referred to as the W-B2 phase, and the matrix with Ni-like structure (fcc). The matrix phase (denoted NiCo2W in what follows) is a solid solution of Co in Ni with a small addition of W which diffused into the NiCo-based matrix.

It was found by neutron diffraction [36] that the original as-sintered sample consisted of fine-grained W-B2 phase surrounded by a coarse-grained NiCo2W matrix. Both cold and warm rotary swaging caused fragmentation of the NiCo2W and resulted in formation of fine-grained matrix microstructure.

Swaging at room temperature (sample W_A) resulted in a decrease of the agglomerate size and in shortening of the inter-agglomerate distances (Figure 2c), as well as in deformation of the agglomerates to a non-spherical shape. Swaging at room temperature also resulted in the radial flow tendency imparted by the radial swaging force component which is visible on the cross-sectional cut. Swaging at 900 °C (sample W_B) also imparted a decrease of the agglomerate size and shortening of the inter-agglomerate distances (Figure 2d), although not so pronounced as in the case of cold swaged sample. The radial plastic flow of the swaged material was also less visible.

### 3.2. Data for Texture Determination

**Figure 3:**
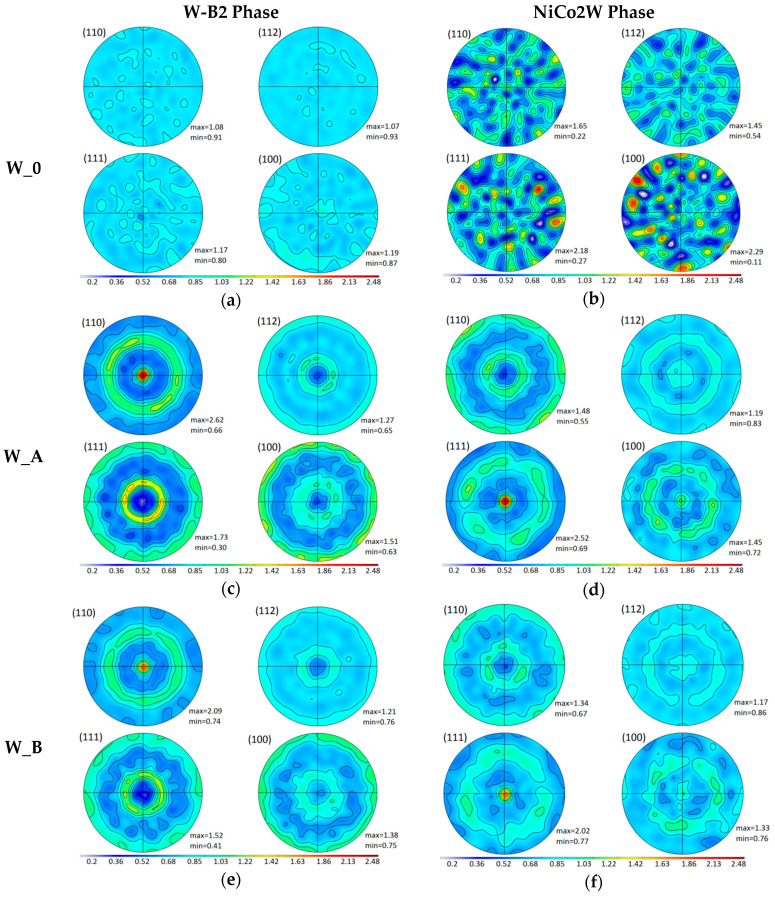
The measured pole figures for all samples (marked on the left, W_0 (**a**,**b**); W_A (**c**,**d**); W_B (**e**,**f**)) and both phases (marked on the top, B2 (**a**,**c**,**e**); NiCo2W (**b**,**d**,**f**)) and all measured reflections. The intensity scale is identical for all graphs. The swaging axis (z) was perpendicular to the figure plane.

The texture measurement was carried out at the neutron diffractometer in all three sample bars for both present phases W-B2 and NiCo2W using reflections 110, 112, 111, 100. From the measured data, the pole figures were calculated using JTEX software [37]. The obtained pole figures are displayed in Figure 3. The individual pole figures in Figure 3 are plotted in the same scale for all three samples and both phases in order to compare absolutely the texture extent between samples and phases (i.e., not only a qualitative characterization of the preferential orientation).

From the pole figures, it is clear the W-B2 phase within the original as-sintered W_0 sample exhibited no texture. The corresponding pole figure also confirms that there are very large grains of NiCo2W phase in the W_0 bar without any clear preferential orientation.

As the spotty pattern of NiCo2W W_0 pole figure changes to smooth contours in pole figure diagrams of W_A and W_B bars, it is clear that the large grains of NiCo2W were obviously largely refined during the rotary swaging to form fine-grained microstructures within both the W_A and W_B samples.

### 3.3. Stress Differences

**Figure 4:**
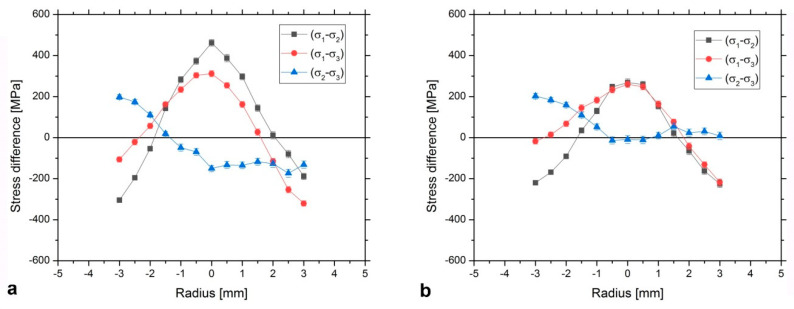
Stress differences for swaged sample bars: W_A (**a**); W_B (**b**).

Stress differences are valuable to identify trends and enable a comparison between both swaged sample bars W_A and W_B. The determined stress differences (*σ_i_*−*σ_j_*) for both samples are shown in Figure 4. We remind that the index 1 stands for axial direction, the index 2 for radial and 3 for hoop directions. Then (*σ*_1_−*σ*_2_) difference means the difference between axial and radial stresses, (*σ*_1_−*σ*_3_) is then the difference between axial and hoop stresses, and finally (*σ*_2_−*σ*_3_) denotes the difference between radial and hoop stresses.

As can be seen, the (*σ_1_*−*σ_2_*) difference was ≈200 MPa larger for the W_A than for the W_B sample at *r* = 0 (where *r* denotes the distance from the center of the cylindrical bar), and the distinction decreased to ≈−100 MPa at the radius of −3 mm. The (*σ*_1_−*σ*_3_) difference was ≈50 MPa higher at *r* = 0, and by ≈100 MPa smaller at *r* = +3 mm for the W_A sample, when compared to the W_B one. The (*σ*_2_−*σ*_3_) profile exhibited the most significant difference at the right side of the sample bar, i.e., along the positive values of the measured radius, where the W_B sample exhibited ≈0 MPa, while the value was ≈−150 MPa for the W_A sample.

The (*σ*_1_−*σ*_2_) profile can be considered roughly symmetric for both the W_A and the W_B sample bars. This symmetry was not observed for the other stress differences in both the W_A and W_B samples. Regardless of the particular analyzed stress difference and sample, the sample center exhibited either a maximum or a minimum value of the particular stress difference, even in spite of the asymmetry observed for the (*σ*_1_−*σ*_3_) and (*σ*_2_−*σ*_3_). It is also notable that (*σ*_2_−*σ*_3_), i.e., the radial-hoop stress difference, differs clearly from zero at the sample center for the W_A sample, although the sample is of cylindrical symmetry after the rotary swaging.

### 3.4. Mechanical Properties

The mechanical properties of the as-sintered bar as well as the swaged bars determined via tensile testing were already published in [36] (as-sintered sample and final passes of the rotary swaging), and in [4] (including initial passes of the rotary swaging). Here, the main results of the testing are summarized. The lowest ultimate tensile strength (UTS) of approximately 860 MPa was recorded for the W_0 sintered bar. On the other hand, this sample bar exhibited substantial plasticity of almost 20%. The high plasticity of the sintered material state is primarily provided by the matrix, since the sintering temperature is sufficiently high to result in a recrystallized structure for the NiCo phase. Swaging at both the room and elevated temperatures resulted in concurrent UTS increase and plasticity decrease, except for the W_B sample swaged via a single pass, the plasticity for which slightly increased when compared to the sintered state. The final swaging resulted in the increase in UTS up to more than 1800 MPa for the W_A swaged bar. The UTS for the W_B swaged bar was approximately 100 MPa lower than for the W_A. Nevertheless, the plasticity was higher for this sample bar. The significant increase in the UTS after the rotary swaging is primarily caused by the deformation strengthening which introduced a significant accumulation of dislocations in the NiCo2W phase. The dislocation accumulation resulted in a large micro-strain increase [36] connected to the residual stresses of type III [38]. The overall strengthening in the cold-swaged bar is higher since the increased swaging temperature leads to dynamic relaxation processes within the matrix which enhance plasticity but also lowers the UTS.

## 4. Discussion

While the original as-sintered sample W_0 exhibited no texture, the pole figures displayed in Figure 3 show strong deformation texture for both W_A and W_B samples. On the other hand, there is no visible special pattern in preferential grain orientation around the sample axis: The intensity exhibits always circular symmetry, which indicates a random radial orientation of crystallites and thus fiber texture. It means that the elliptical cross section of the original as-sintered sample bar is not reflected anyhow in the grain preferential orientation after rotary swaging.

**Figure 5:**
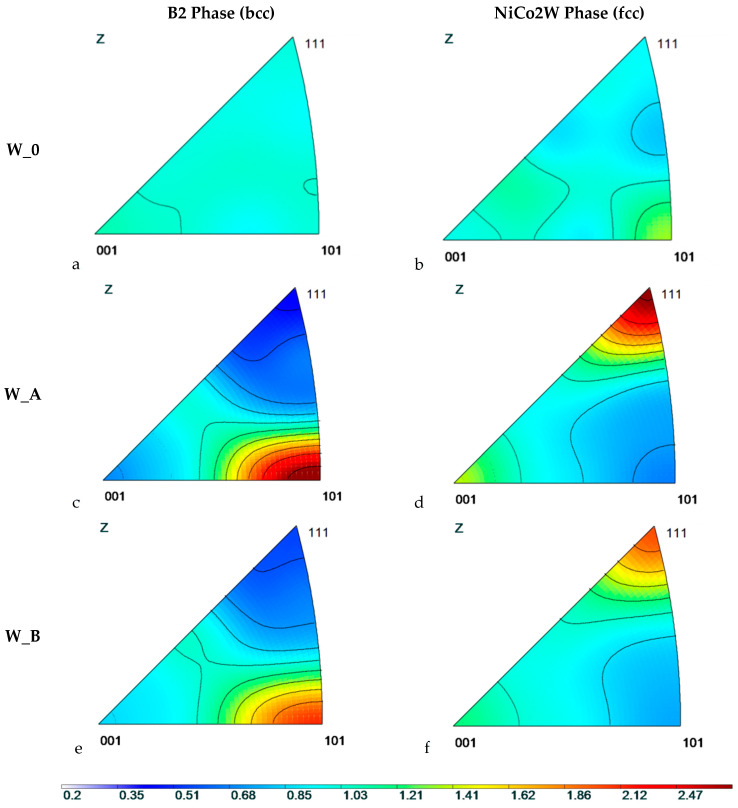
The inverse pole figures for all the samples (marked on the left: W_0 (**a**,**b**); W_A (**c**,**d**); W_B (**e**,**f**)) and both phases W-B2, NiCo2W (marked on the top: B2 (**a**,**c**,**e**); NiCo2W (**b**,**d**,**f**)) for the direction along the sample-bar axis (*z* or direction 1). The intensity scale is identical for all graphs.

The inverse pole figures were calculated from the measured pole figures. The most important pole figures are those measured along the sample-bar axis (z direction, denoted also by index 1 in this paper) which was the symmetry axis of the plastic deformation, i.e., the swaging axis. The results for z direction can be seen in Figure 5.

The inverse pole figures confirm that the grains within both the W-B2 and NiCo2W phases in the as-sintered W_0 sample bar did not exhibit any prevailing preferential orientation. An indication of small preferential orientation (fiber <101>) in NiCo2W phase (see Figure 5) is inconclusive as the extremely large grain, estimated to 0.2–1 mm size [36], was detected in NiCo2W phase. Therefore, the inverse pole figure is deduced from only a very small number of grains, as can be seen from the spotty pole figure of W_0 bar in Figure 3.

On the other hand, a strong deformation texture was formed in both phases after rotary swaging. The textures in both samples W_A and W_B are qualitatively the same: W-B2 phase of tungsten (bcc structure) is textured with crystallographic orientation <101> preferentially along the sample bar axis after the rotary swaging while NiCo2W phase (fcc structure) is textured with the crystallographic orientation <111> preferentially along the sample bar axis (i.e., swaging direction). Development of <101> fiber texture after severe deformation in W-B2 phase of THAs was reported previously. For example, Ekbom et al. [39] observed <101> texture in a heavy alloy (90W, 7Ni, 3Fe) prepared by sintering and deformed either in a tensile testing machine or by extrusion. Gong et al. [20] determined <101> texture in tungsten phase of fine-grained 93W–4.9Ni–2.1Fe–0.03Y (wt.%) alloy after hot extrusion. Nevertheless, neither [39] nor [20] reported a crystallographic texture of the matrix phase in the THA composite structure, which obviously can be significantly different, and neither compared the texture of samples processed at different temperatures under otherwise the same conditions.

For bcc metals, <101> fiber texture is generally predicted after uniaxial tension or wire drawing [40]. Our observation for W-B2 phase corresponds well to this typical pattern. In the sense of preferential orientation of W-B2 agglomerates, rotary swaging is thus similar to uniaxial tension deformation.

Nevertheless, for fcc metals (as is our NiCo2W phase) the typical fcc tension texture is a mixed <001> and <111> fiber texture [40,41]. Although the proportion between these two fibers depends on the stacking fault error, <001> fiber should still be forming around 35% of the volume fraction for nickel [42]. Our observation deviates from this typical behavior as the <001> fiber texture is largely suppressed (see Figure 5) and <111> fiber texture fully dominates the preferential orientation of the matrix. Suppression of the <001> component is most probably due to the interaction of NiCo2W phase with the second component of the investigated metallic composite, i.e., with the W-B2 phase. Another explanation could lie in the rotary swaging technique itself, which would mean that this technique is capable to form a non-standard texture in fcc metals. It is, nevertheless, sure that the temperature plays no role in this pattern, as the <001> fiber was suppressed with respect to the <111> fiber in both samples swaged at RT and at the elevated temperature.

Although the textures in both samples W_A and W_B are qualitatively the same, there is a significant difference between the sample W_A and the sample W_B: the W_A sample (deformed at room temperature) exhibits much stronger texture than W_B (deformed at 900 °C) for both present phases (W-B2 as well as NiCo2W). It can be seen in the inverse-pole-diagram figure (Figure 5, see the color scale) as well as in the Table 1 showing the relative maximum intensities in the pole figures. This difference is highly probably caused by the different deformation temperatures for the two samples. As the weaker texture formed during the deformation at 900 °C, it indicates that also other than primary deformation mechanism for the bcc phase (W-B2) as well as for the fcc phase (NiCo2W) were, to a certain extent, activated. (Reminder: the primary deformation mechanism for plastic deformation in fcc crystals is the crystallographic slip on the {111} crystallographic planes along the <110> crystallographic directions, while it is the slip on the {110} crystallographic planes along the <111> directions for bcc crystals.) The secondary deformation mechanisms were most probably enabled by a substantially larger diffusion of atoms at high temperature. These additional deformation mechanisms lower the amount of crystallite rotation caused by the movement of dislocations on primary slip planes.

The differences in the determined residual stresses between both the W_A and W_B samples can be also ascribed to the different deformation temperatures. Before discussing the details of the individual stress differences, the von Mises equivalent stress formula is employed for comparison of the overall level of the residual stresses in both investigated bars.

Von Mises equivalent stress *σ_VM_* determines a limit, above which yielding starts, and is thus also termed the maximum distortion criterion. Under multi-axial loading conditions, it is used to predict yielding of materials. A larger value of von Mises equivalent stress implies that the material is closer to the yield point. The *σ_VM_* stress is determined by the expression (in case when the stresses are calculated in the orthogonal directions)

(4) $$\sqrt{\frac{{\left(\sigma_{1}-\sigma_{2}\right)}^{2}+{\left(\sigma_{1}-\sigma_{3}\right)}^{2}+{\left(\sigma_{2}-\sigma_{3}\right)}^{2}}{2}}$$

When the same formula is used with the measured residual-stress differences, the obtained resulting stress level constitutes a simple single parameter providing information about the overall residual stress level in the particular location in the bar, and can be also used for determining a region where the plastic deformation, and consequently the texture, was the largest. It also enables an overall comparison of the residual stress levels in W_A and W_B samples.

**Figure 6:**
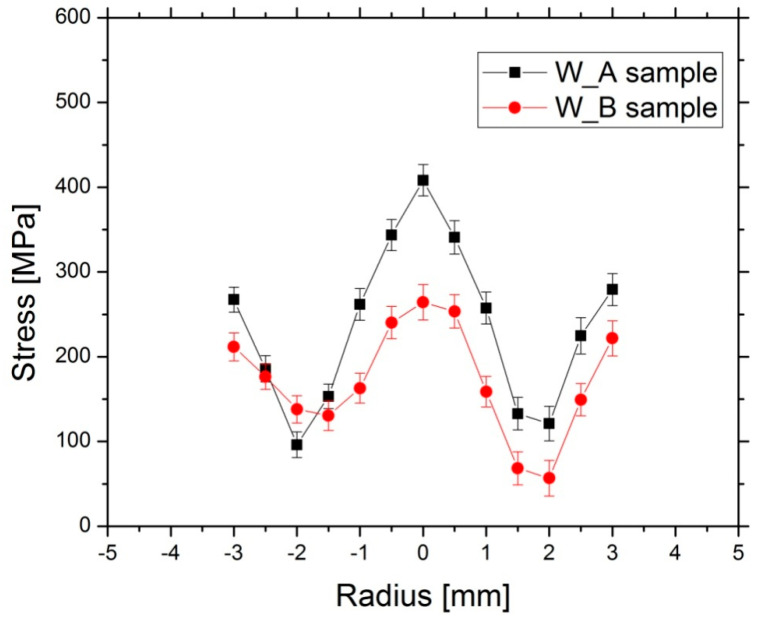
The stress calculated using von Mises formula (4) from the measured differences of the stresses for W_A and W_B samples.

By applying this approach to our measured residual stress differences for W_A and W_B samples, the profiles which are shown in Figure 6 are obtained. The maximum stresses of ≈400 MPa and of ≈250 MPa are observed at the center of the bar for the W_A and W_B samples, respectively, while minima are reached at *r* = ± 2 mm. Obviously, the point closest to yielding is in the center of each bar. This is qualitatively the same for the sample deformed at RT and for the sample deformed at 900 °C. It is interesting as the applied deformation force was at the surface. Nevertheless, we must take into account that the range in which the residual stresses were measured did not span the full cross section of the bar. There was no measurement near the surface region where—in principle—the residual stresses could be still higher. More detailed considerations about the residual stresses are to be carried out in order to estimate the residual stresses near the surface and to calculate the stress value according to the von Mises-like formula in that locations.

Nevertheless, if we assume only the measured region, the stress level is larger (up to 150 MPa larger) at the center of the bar (between *r* = −1 mm and *r* = 1 mm) for the W_A when compared to the W_B sample, while they are only ≈50 MPa larger closer to the outer surface. Since the larger residual stress level indicates that there were larger stresses during the deformation process at that particular region, it is possible to infer that a larger plasticization occurred in the sample center. Then, consequently, a stronger texture was built in the sample center. This cannot be deduced from the texture measurement itself (described in the previous section) as the texture measurement was carried out with the gauge volume containing the whole cross section of the bar and local variations cannot be thus distinguished. In this way, it is possible to correlate the observed texture differences between both W_A and W_B samples and the residual stresses developed during the rotary swaging process: W_A sample exhibiting stronger texture also exhibits higher residual stress level, mainly in the core of the bar.

It can be deduced from the shape of the overall stress level (Figure 6) that the qualitative character of the residual stresses distribution is the same for both samples, i.e., the cold as well as hot swaged, but the overall level is lower for W_B deformed at HT. The lower level must be connected with the deformation at high temperature (HT). At HT, yield stress is lower than at RT. Residual stresses created during swaging at HT cannot be higher than yield stress at that temperature (they would cause yielding otherwise). Therefore, the residual stresses formed at HT (i.e., before cooling) are lower than the residual stresses formed during deformation at RT in the cold swaged sample bar. Additionally, restoration phenomena (which are likely to be activated at higher temperatures, dependent on diffusion of atoms) take place at HT, which further lower the residual stresses formed during swaging at high temperature. Such restoration processes are hindered during the deformation at room temperature.

The cooling obviously did not change the overall picture of the residual stresses, as the character of residual stresses is the same for W_B sample after cooling from high temperature and for W_A sample (for which no cooling took place). A typical cooling stress profile is smooth and parabolic [43], as well as the (*σ*_1_−*σ*_2_) difference [44]. If cooling induced stresses played an important role, the (*σ*_1_−*σ*_2_) difference would be positive close to the outer surface. However, it is observed to be ≈−200 MPa at *r* = ± 3 mm (Figure 4b) in this study. The cooling could hypothetically play a role in the asymmetry of the overall stress level in the W_B sample in case the cooling of the bar proceeded axially non-symmetrically. It can be seen in Figure 6 that the stress is nearly symmetric for W_A sample (RT swaging) while the minima are slightly asymmetric for W_B bar (900 °C swaging followed by cooling to RT). However, stresses developed during cooling are expected to be roughly symmetric in all directions in a circular cross-section bar. Further, a detailed investigation of the individual differential stresses (*σ*_1_−*σ*_3_) and (*σ*_2_−*σ*_3_) rather points at another cause of the asymmetry.

The profile asymmetries depicted in Figure 4, noticeably the largest for the hoop stresses, were highly likely the consequence of the elliptical cross section of the original sintered bar. The initial non-circular sintered sample cross-section can finally contribute to a non-symmetric internal stress distribution during the deformation process, provoking the asymmetry in the stress differences profiles containing the *σ*_3_ stress, which is manifested mainly by the (*σ*_2_−*σ*_3_) difference. It should also be noted that—as a result of the experimental setup for residual stresses measurement—the elongated gauge volume is smoothing the real stress differences. As a consequence, the presented stress differences are a bottom limit of the real differences. Due to these reasons, the determined (*σ*_2_−*σ*_3_) stress difference is not equal to zero at the center of the W_A sample bar.

The differences in the stresses between the W_A and W_B samples (see Figure 4) were the largest for the (*σ*_1_−*σ*_2_) differential stress. The observed (*σ*_1_−*σ*_2_) difference is always positive at the center of the sample bar and negative at the radius *r* = ± 3 for both the studied W_A and W_B samples, suggesting that the axial stresses are always larger at the center of the bar and smaller close to the outer surface when compared to the radial stresses. Assuming the radial stress (*σ*_2_) must be close to zero at *r* = ± 5 mm to comply with the boundary condition, it is straightforward to assume that axial stresses (*σ*_1_) are compressive close to the outer surface (see Figure 4). A further condition is that the axial stresses integrated over the cross section are self-equilibrated-which requires that tensile axial stresses develop at the center. At the same time, the (*σ*_1_−*σ*_2_) difference changes sharply, more noticeably for the W_A sample. Therefore, it is reasonable to deduce that the radial stress is negative at the center of the sample (approaching to zero when close to the outer surface) in order to produce this observed sharp (*σ*_1_−*σ*_2_) profile variation as a function of *r*.

Another interesting point is if the measurement would have been performed in the same cross-section but in another diametrical line. This study would require the measurement of absolute stresses, i.e., the presence of a reliable stress-free reference. Nevertheless, since the stress differences involving the hoop component always exhibit large asymmetries, the hoop-stress magnitude is expected to be dependent on the measured line while the axial and radial components are supposed to be less affected.

The more extensive stress analysis with employed complex equilibrium condition is to be published in a separate article.

An obvious correlation between the texture strength and UTS can be observed: the higher UTS corresponds to the more pronounced texture in W_A sample with respect to W_B one. The larger texture strength of NiCo2W phase with <111> fiber type in the sample swaged at RT means that more matrix crystallites are oriented in this way than in the sample swaged at 900 °C. As the yield strength in fcc crystals is highest just for the <111> crystallographic direction [45], larger yield stress as well as UTS can be expected at the sample swaged at RT. Nevertheless, another cause for higher yield stress and UTS can be the observed larger dislocation density [36] after rotary swaging, which was observed in the sample swaged at RT.

## 5. Conclusions

The study was focused on the investigation of the effects of cold and warm rotary swaging regimes on the microstructure characteristics of a WNiCo tungsten heavy pseudoalloy.

The analysis confirmed that the as-sintered sample consisted of fine-grained W-B2 tungsten agglomerates and coarse-grained NiCo2W matrix. This finding can be attributed to the high sintering temperature promoting significant grain growth within the NiCo2W matrix. No preferential crystallographic orientation can be deduced in the as-sintered structure, neither in W-B2 agglomerates, nor in NiCo2W matrix.

During rotary swaging, the large grains of NiCo2W fractioned to form a fine-grained microstructure. A strong texture was formed in both NiCo2W and W-B2 phases after rotary swaging. The texture type is different for these two phases, which was expected as the W-B2 phase is bcc while the NiCo2W phase is fcc. The W-B2 phase formed fiber texture with the <101> parallel to the swaging axis, while <111> fiber was parallel to the swaging axis for NiCo2W phase. For W-B2 phase, it is the typical behavior of uniaxially deformed bcc metal. However, NiCo2W phase texture is non-typical, as <001> fiber of the usually double-fiber texture is largely suppressed.

Although the textures in both rotary swaged samples (20 °C and 900 °C) are qualitatively the same, the cold-swaged bar exhibited substantially stronger texture for both phases. Obviously, the different deformation temperatures played a role here. This observation corresponds to the higher measured ultimate tensile strength of more than 1800 MPa for the cold-swaged bar: both the tensile strength and the texture intensity indicate substantial deformation strengthening and hindered restoration during room temperature swaging.

The largest residual stress differences were found for the sample deformed at room temperature. The maximum (*σ*_1_−*σ*_2_), i.e., axial-radial difference, of ≈400 MPa was found at the center of the sample and the minimum of ≈−300 MPa at the radius of −3 mm. The hoop stress distribution was found to be non-symmetrical along the swaging axis for both the swaged bars.

The residual stress profile determined using von Mises-type formula exhibited larger values at the center of the sample bars and minima at *r* = ± 2 mm. The profile is symmetric for W_A bar (despite the observed asymmetries in the individual stress differences, mainly for the *(σ*_2_−*σ*_3_*)* difference), while its minima are non-symmetric for W_B sample. Detailed evaluation of the asymmetries leads to the conclusion that it was likely caused by the elliptical cross-section of the as-sintered bar.

Comparison of the differential stresses and the textures indicates that the texture difference between samples swaged at RT and at 900 °C develops mainly in the inner core of the deformed bars.

## Figures and Tables

**Table 1:** Relative maximum intensities in the pole figures.

Sample Bar	Processing	W-B2 Phase	NiCo2W Phase
W_0	as sintered	1.19	---
W_A	swaged at RT	2.62	2.52
W_B	swaged at 900 °C	2.09	2.02
